# *In vivo* and *ex vivo* study on cell wall components as part of the network in tomato fruit during the ripening process

**DOI:** 10.1093/hr/uhae145

**Published:** 2024-05-24

**Authors:** Nataliia Kutyrieva-Nowak, Agata Leszczuk, Dusan Denic, Samia Bellaidi, Konstantinos Blazakis, Petroula Gemeliari, Magdalena Lis, Panagiotis Kalaitzis, Artur Zdunek

**Affiliations:** Institute of Agrophysics, Polish Academy of Sciences, 20-290 Lublin, Poland; Institute of Agrophysics, Polish Academy of Sciences, 20-290 Lublin, Poland; Department of Horticultural Genetics and Biotechnology, Mediterranean Agronomic Institute of Chania, Chania 73100, Greece; Department of Horticultural Genetics and Biotechnology, Mediterranean Agronomic Institute of Chania, Chania 73100, Greece; Department of Horticultural Genetics and Biotechnology, Mediterranean Agronomic Institute of Chania, Chania 73100, Greece; Department of Horticultural Genetics and Biotechnology, Mediterranean Agronomic Institute of Chania, Chania 73100, Greece; Department of Biomedicine and Environmental Research, Institute of Biological Sciences, Faculty of Medicine, John Paul II Catholic University of Lublin, 20-708 Lublin, Poland; Department of Horticultural Genetics and Biotechnology, Mediterranean Agronomic Institute of Chania, Chania 73100, Greece; Institute of Agrophysics, Polish Academy of Sciences, 20-290 Lublin, Poland

## Abstract

Ripening is a process involving various morphological, physiological, and biochemical changes in fruits. This process is affected by modifications in the cell wall structure, particularly in the composition of polysaccharides and proteins. The cell wall assembly is a network of polysaccharides and proteoglycans named the arabinoxylan pectin arabinogalactan protein1 (APAP1). The complex consists of the arabinogalactan protein (AGP) core with the pectin domain including arabinogalactan (AG) type II, homogalacturonan (HG), and rhamnogalacturonan I (RG-I). The present paper aims to determine the impact of a disturbance in the synthesis of one constituent on the integrity of the cell wall. Therefore, in the current work, we have tested the impact of modified expression of the *SlP4H3* gene connected with proline hydroxylase (P4H) activity on AGP presence in the fruit matrix. Using an immunolabelling technique (CLSM), an immunogold method (TEM), molecular tools, and calcium mapping (SEM-EDS), we have demonstrated that disturbances in AGP synthesis affect the entire cell wall structure. Changes in the spatio-temporal AGP distribution may be related to the formation of a network between AGPs with other cell wall components. Moreover, the modified structure of the cell wall assembly induces morphological changes visible at the cellular level during the progression of the ripening process. These results support the hypothesis that AGPs and pectins are required for the proper progression of the physiological processes occurring in fruits.

## Introduction

Ripening is a postdevelopment process composed of different morphological, physiological, and biochemical changes [1]. It includes a series of changes that alter a green unripe fruit into a red ripe fruit with an appropriate appearance, taste, and soft texture. The softening and textural modifications are due to changes in the composition of polymers and their binding in the cell matrix in fruit tissue [2]. The alterations include modification of the cell wall structure from the degradation of pectin and hemicellulose to the synthesis of new polymers [3, 4]. Fruit ripening is a coordinated developmental programme undoubtedly related to changes in the architecture of the primary cell wall mainly due to softening progression [5]. Recent research has challenged individual cell wall models and suggests that specific components are closely related to each other and have a much more direct impact on defining fruit properties [6,7]. On the other side, the complexity of the fruit tissue provides opportunities to improve mature fruit characteristics. Currently, it is desirable to select varieties whose fruits are tolerant to cracking, epidermal shrivelling, delay acceleration of softening progression, characterized by higher nutritional value, and resilient to climate change. Also, the analysis demonstrated that fruit disruption rate is associated most significantly with cellulose content and its interaction with other components [8]. It is also necessary to emphasize that the ripening programme is coordinated by the effects of epigenetic modifications, transcription factors, and plant hormones [9]. Moreover, studies at the molecular level show that biochemical changes coordinated by proteins entangled in signal transduction and membrane interactions also influence fruit quality. Therefore, in this context, structural studies such as subcellular localization of a protein and general bio-imaging are essential steps toward understanding its activities [10]. Only focusing on explaining the connections with cell wall metabolism, hormonal activity, and genetic modifications in fruit ripening allows for a complete description of the programmes taking place in fruit.

The structure of the cell wall is investigated in ongoing studies, and the functions of particular polysaccharides and proteins are being debated [11, 12]. The cell wall is a complex assembly consisting mainly of cellulose, hemicelluloses accompanied by pectins, and proteins [4, 13]. Cellulose is composed of a linear chain of 500–7500 of β-(1 → 4)-d-glucose (Glc) monomers assembled by hydrogen bonding to constitute microfibrils [14]. Also within the cell wall, there are numerous matrix glycans, known as hemicelluloses, i.e. a group of polysaccharides composed of Glc, xylose (Xyl) or mannose (Man), and xyloglucan (XyG) [15]. It is well known that XyG binds to the surface of microfibrils forming a network with cellulose [16]. Structural changes and rearrangements of linkages in hemicellulose and cellulose disrupt the middle lamella. The disruption of xyloglucan and other glycans affects the ripening progress by inducing the softening process in fruits [17]. Other hemicellulosic polymers, i.e. xylans with β-(1 → 4)-xylosyl backbone cross-linking polysaccharides in the structure of the cell wall, also affect cell wall integrity and architecture [18–20]. As linear glycan chains of β-(1,4)-linked mannose residues [18], mannans play a role in the softening and degradation of the cell wall during fruit ripening [21]. Another heterogeneous group of structural cell wall components are pectins composed of α-(1,4)-linked galacturonic acids (GalA). They are divided into three polymers: homogalacturonan (HG), rhamnogalacturonan I (RG-I), and complex rhamnogalacturonan II (RG-II) [18, 22, 23]. HG is an abundant linear pectic subtype, representing 55%–70% of pectin. It is classified according to the esterification degree into high-methoxylated HG and low-methoxylated HG [24, 25]. RG-I and RG-II are pectin subtypes mainly found in primary plant cell walls. RG-I is composed of GalA and rhamnose (Rha) residues with side chains of arabinan, arabinogalactan, and galactan [26]. RG-II is composed of glycosyl residues comparable to the repeating GalA and Rha residues in RG-I. It has a highly conserved and complex structure with approximately ten different saccharides in side chains [27].

Modifications of carbohydrate chains have a significant effect on the ripening process [4]. The cell wall degradation is associated with pectin solubilization and depolymerization and a decrease in the number of pectic side chains [28, 29]. The HG depolymerization reduces intercellular adhesion, thereby having an impact on turgor and contributing to tissue softening [30–32]. In turn, the degradation of HG molecules during the ripening process leads to the disassembly of the links between cellulose and hemicellulose [23, 25]. Also, RG-I and RG-II are involved in cell-to-cell signalling and the formation of the pectic network, and degradation of these pectins exerts an effect on the cell wall mechanical properties [33, 34].

Furthermore, cell wall proteins (CWPs) are important structural and functional constituents of the plant cell walls [13]. Proteins are categorized as hydroxyproline-rich glycoproteins (HRGPs) with arabinogalactan proteins (AGPs), glycine-rich proteins (GRPs), and proline-rich proteins (PRPs) [18, 35, 36]. They have numerous functions in the plant cell, including signal transduction, cell-to-cell interactions, cell wall-to-plasma membrane interactions, and provide additional support and rigidity to the cell wall [13, 37]. Extensins, belonging to HRGP, are involved in plant cell development as regulators of cell wall expansion, chains binding the cell wall with the plasma membrane, and polymers forming the cellular architecture [38, 39]. AGPs are unusual proteoglycans localized between the cell wall and plasma membrane and have a variety of biological functions, whose basis has not been definitively identified [40]. They have a role in cell differentiation and cellular communication [41]. Also, AGPs probably have an impact on cell division, plant growth and development, reproductive processes, and programmed cell death [42]. Our previous research has shown that AGPs may have an effect on anatomical and morphological alterations in the fruit cell wall during the ripening process. In the last stages of ripening, we noted a decrease in the number of AGPs associated with disruption of the native cell wall structure [43, 44].

**Figure 1:**
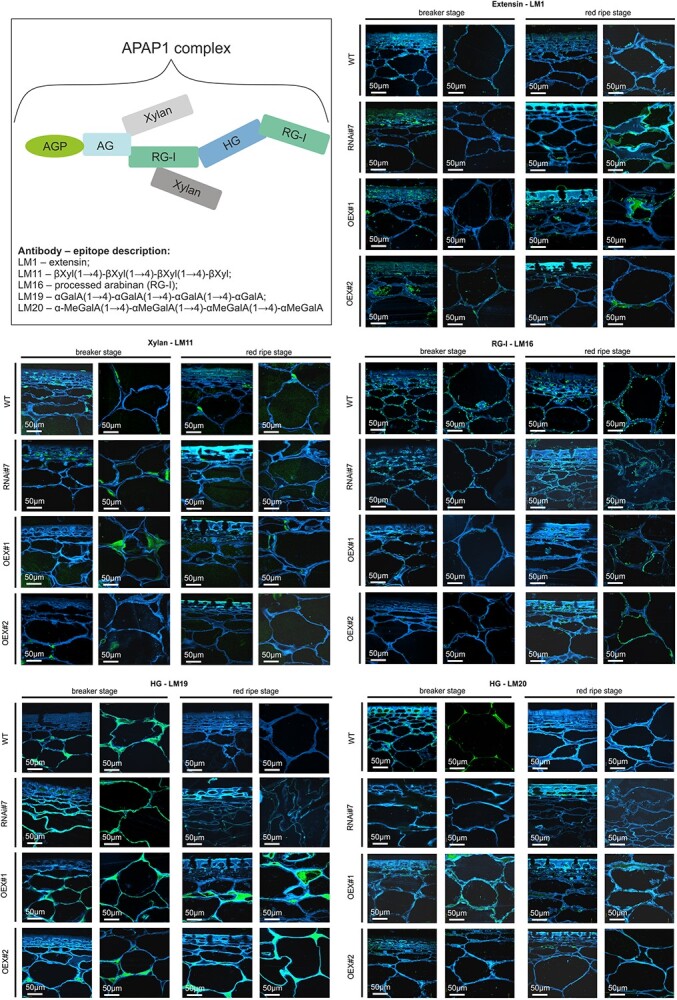
Hypothetical and simplified structure of the APAP1 [45, 75] with a description of the antibodies used. The spatio-temporal localization of extensin (LM1), xylan (LM11), rhamnogalacturonan type I (LM16), low methyl-esterified homogalacturonan (LM19), and high methyl-esterified homogalacturonan (LM20) in the fruit tissue of the WT plants and transgenic lines RNAi#7, OEX#1, and OEX#2 in different ripening stages. Abbreviations: AG, arabinogalactan; RG-I, rhamnogalacturonan I; HG, homogalacturonan Bars 50 μm for all images.

To emphasize the importance of the cell wall, it is necessary to mention that cell wall components interact with each other and form complex extracellular networks that control cell growth and help maintain cell shape. Generally, non-covalent interactions are found between pectic HG domains, cellulose chains, and cellulose with xylan and xyloglucan [20, 23, 45]. In turn, covalent interactions are found between HRGPs, pectin RG-I and RG-II monomers, and polysaccharides with lignins [23, 45, 46]. In the cell wall integrity context, AGPs play an important role, as they form the system of polysaccharides and proteoglycans in the cell wall–plasma membrane continuum [47] by creating the arabinoxylan pectin arabinogalactan protein1 (APAP1) [45]. The APAP1 is an AGP core with the classical AG attached. The APAP1 pectin domain includes HG and RG-I, in which short HG oligosaccharides are located around RG-I (graphic scheme in Fig. 1). In addition, the APAP1 contains arabinoxylan, which is attached to the Ara residue from AG and to Rha in RG-I [12, 45]. The synthesis of the APAP1 probably occurs in the extracellular matrix by binding pectin and arabinoxylan glycans to AGPs [45,48]. The components of the APAP1 are synthesized in different cellular compartments. For example, AGP synthesis proceeds in several stages, starting with the synthesis of the protein backbone, the glycosylphosphatidylinositol (GPI) anchor in the endoplasmic reticulum, and the sugar chains in the Golgi apparatus and ending in the formation of the AGP molecule in the space between the cell wall and plasma membrane [35, 47]. Pectin and xylan synthesis proceeds in the Golgi apparatus [34, 45]. Scientific reports have so far confirmed that changes in the pathways for the synthesis of the particular components of the APAP1 can cause disruptions in its structure and function. Interestingly, changes in the binding between the components also affect the cell wall structure, and these alterations are associated with the shape, turgor, and size of fruit cells [4, 49, 50]. These connections provide strength and stiffness to the cell walls, which have an influence on the fruit structure during the developmental programme, ripening process, and senescence in postharvest storage [51]. For example, ripening is correlated with alterations in the fruit texture and firmness, i.e. a process of swelling of the middle lamella [52], which is related to changes in the negative charge and structure of pectin polymers [51]. The research conducted by Castro and coworkers revealed an inverse correlation between fruit firmness and depolymerization of cell walls [4].

Another feature emphasizing the important role of the connections between the cell wall components is the binding of calcium ions. Currently, it has been proved that GalA residues from pectins and GlcA residues from AGPs bind Ca^2+^ and form calcium bridges between cells [48, 53–56]. The hypothetical molecular basis of the mechanism of AGP action is based on calcium cross-linking. AGPs take part in physiological processes by interacting with Ca^2+^, followed by the release of Ca^2+^ [57, 58]. This was confirmed by a study on β-glucuronyltransferase mutants (GlcAT) where the depletion of Ca^2+^ binding affected the stability of the cell wall–plasma membrane continuum, which stopped plant development [59, 60]. Other investigations of GLCAT14A, GLCAT14B, and GLCAT14C mutants showed that β-glucuronyltransferases 14 (GT) were accountable for the binding of GlcA residues to AGPs [61]. Besides, the decrease in GlcA in the double and triple mutants affected cell differentiation and plant growth. Most likely, these changes are related to reduced Ca^2+^ influx into particular cells [60, 61]. Thus, it can be concluded that, as part of AGP and pectins in the APAP1, GlcA has an effect on cell wall integrity and cell-to-cell signalling via Ca^2+^ binding [53, 56]. It is well-known that calcium ions are implicated in biological processes, including cell division and elongation, and stabilization of the cell wall [62]. Moreover, calcium helps maintain the structural integrity of the cell wall and is a cofactor for various enzymes involved in fruit ripening, such as pectin methylesterase (PME) and polygalacturonase (PG) [33, 63]. The structural integrity is related to the ability of calcium ions to form cross-links between pectin molecules, which helps strengthen the cell walls and prevent the breakdown of cells during ripening, maintaining the firmness of fruit [63, 64]. Moreover, calcium ions interact as single signalling molecules or co-interact with plant hormones, such as abscisic acid (ABA), to regulate biochemical changes associated with ripening [65]. In addition, calcium ions control the synthesis and breakdown of various compounds, such as pigments, flavour compounds, and aroma volatiles. A study conducted by Xu and coworkers demonstrated that calcium ions have the ability to modulate fruit polyphenolic biosynthesis, e.g. anthocyanins in strawberries, whose content increases significantly as the fruit ripens [66].

Combining all the above-mentioned features of the cell wall, structure of components, connections between them, and the calcium ion binding ability as well as their impact on the ripening process, we aim to determine the effect of a disturbance in the synthesis of one constituent on the integrity of the cell wall at the molecular level and, consequently, at the level of the entire fruit organ. In our previous studies on modified tomato fruits, we focused on analysis of the effect of modification of *SlP4H3* expression encoding the P4H3 enzyme, which is responsible for the posttranslational modification of AGP, on the presence of AGP in the cell wall [67]. The physiological role of *SlP4H3* in developmental programmes such as fruit ripening and abscission was well demonstrated in silencing and overexpression lines [67–69]. In these *SlP4H3* lines, changes were observed in the spatio-temporal AGP distribution in the red ripe fruit pericarp tissue as well as disruption of the AGP molecular structure and disturbance of the native AGP structure [67]. Moreover, silencing of *SlP4H3* resulted in a delay of overripe fruit abscission progression [69] and alterations in the position of the flower abscission zone [68]. In addition, the silencing of two tomato P4Hs, SlP4H5 and SlP4H7, by VIGS (Virus-Induced Gene Silencing) indicated a delay in ripening progression [7].

In our previous paper, we described that this modification affected the amount and spatio-temporal pattern of distribution of AGPs during the ripening process. We noted changes that led to disruption of the whole-cell wall structure [67]. Therefore, in the current work, we have tested the effect of this AGP modification on the APAP1 for the first time. The purpose of the present work is to identify changes in the content and distribution of other cell wall constituents during the fruit ripening process in transgenic lines with modified *SlP4H3* gene expression.

## Results

### Localization of cell wall components at the cellular level

**Figure 2:**
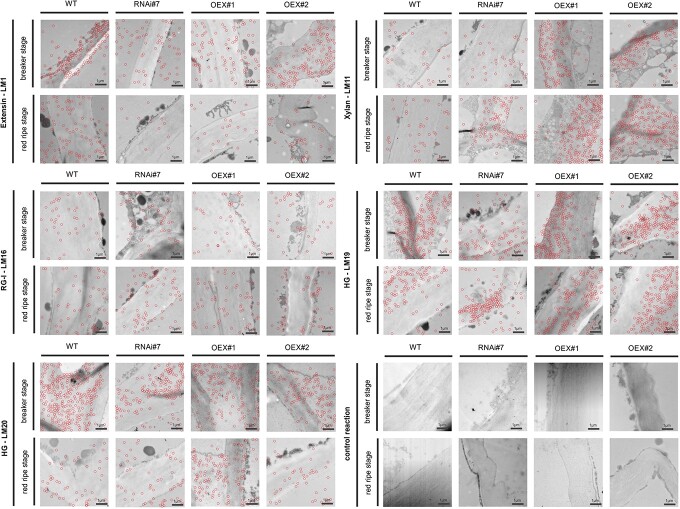
Immunogold labelling distribution of extensin (LM1), xylan (LM11), rhamnogalacturonan type I (LM16), low methyl-esterified homogalacturonan (LM19), and high methyl-esterified homogalacturonan (LM20) in the fruit tissue of the WT plants and transgenic lines RNAi#7, OEX#1, and OEX#2 in different ripening stages. Abbreviations: RG-I, rhamnogalacturonan I; HG, homogalacturonan. Control reaction with lack of incubation with primary antibody. Bars 1 μm for all images. Gold particles marked with red circles.

Fruit visualization at the tissue level allows finding differences in morphology between WT and transgenic lines. The tissues of the modified fruits were more damaged in the RR stage than the tissue of the WT fruits. Moreover, the cell walls in the OEX#1, OEX#2, and RNAi#7 lines after the ripening process were more disturbed with visible interrupted continuity and excessive swelling (Fig. 1). Furthermore, the microscopic studies allowed the determination of the localization of the cell wall components, constituents of the APAP1, and changes in their distribution during the ripening process in the WT fruits and in the transgenic lines OEX#1, OEX#2, and RNAi#7. The use of specific antibodies and the CLSM observations revealed that the modification associated with the changes in the expression of the *SlP4H3* gene affected the assembly of extensin (LM1), xylan (LM11), rhamnogalacturonan (LM16), low methyl-esterified homogalacturonan (LM19), and high methyl-esterified homogalacturonan (LM20) in the cell walls. Representative photographs with immunocytochemical labelling are presented in Fig. 1 (cellular localization) and Fig. 2 (subcellular localization).

The analysis of immunocytochemical labelling with LM1 demonstrated the typical presence of extensin epitopes across the cell wall–plasma membrane continuum. In addition, the occurrence of single conglomerates of the LM1 epitope in the cytoplasm close to the border with the plasma membrane was detected. Moreover, high amounts of extensin epitopes were detected in the cells of the epidermal layer in the BR stages of all the examined lines. The ripening process resulted in a decrease in the number of extensin in the cell wall–plasma membrane continuum. In the RR stage, an increased number of epitopes were observed in the cytoplasm in all the tested lines. Most probably, the localization was associated with degradative processes in the cell wall and the release of previously bound epitopes. The comparison of fruit WT and transgenic lines at each ripening stage showed a significantly higher fluorescence signal in sections from fruit with the overexpression of the *SlP4H3* gene. Also, in the BR stage, the presence of a bigger accumulation of epitopes in the cytoplasm was observed. There were no noticeable differences in the abundance of LM1 epitopes between fruits with the silenced expression of the *SlP4H3* gene and WT.

The immunofluorescence labelling with the LM11 antibody localized the epitopes of xylan in the corners and at cellular junctions in fruit tissues of WT. In the case of all the transgenic lines, the immunolabelling analyses showed a slight increase in fluorescence intensity and revealed an increasing abundance of xylan epitopes during the progress of the ripening process. In addition, an increase in the number of groups of the LM11 epitope was observed near the plasma membrane.

The fluorescence signal after the immunofluorescence reaction with the LM16 antibody was quite weak and was mainly noted along the plasma membrane in association with the cell wall. The ripening process did not affect the presence of the epitope, and similar amounts of the LM16 epitope were observed in the BR and RR stages. The immunofluorescence intensity in the lines with the *SlP4H3* gene overexpression was similar to that in WT. However, the changes in the expression of the *SlP4H3* gene caused higher dispersion of the RG-I epitope within the plasma membrane. In line with the silenced expression of the *SlP4H3* gene, the LM16 epitope was visualized as single larger aggregations rather than a line at the cell wall periphery.

The immunofluorescence analysis with the LM19 and LM20 antibodies recognizing homogalacturonan with various esterification levels revealed the most considerable differences as a result of the alteration in the *SlP4H3* expression. Low methyl-esterified HG (LM19 epitope) in the fruit tissue in the different ripening stages was distributed according to specific spatial patterns in corners and at cellular junctions in the BR stage. The ripening process resulted in a decreased amount of the LM19 epitope forming single dots in the intercellular area. In the fruit tissue with the overexpression of the *SlP4H3* gene, significantly higher fluorescence intensity was observed compared to WT in both the first and last stages of the ripening process. In turn, the silencing of the *SlP4H3* gene caused the occurrence of HG epitopes without characteristic labelled corners. In addition, in the fruits with the silenced expression of the *SlP4H3* gene, a decrease in fluorescence intensity was found from the beginning of the ripening process.

The immunofluorescence reaction with LM20 antibody which recognized high methyl-esterified HG allows discrimination between high- and low-esterified HGs. In the WT fruits, the LM20 epitope was labelled in the cellular junctions and corners, and the immunofluorescence decreased with the ongoing ripening process. However, no significant changes in the immunofluorescence intensity were observed in the transgenic lines compared to WT. At the beginning of the ripening process, a slightly reduced amount of HG epitopes was identified in the fruits with the silenced expression of the *SlP4H3* gene; they were present as single dots in the fruit tissue within the cell wall. In the RR stage, the LM20 epitopes in the fruits with the overexpression of the *SlP4H3* gene were detected as single conglomerates.

### Localization of cell wall components at the subcellular level

**Figure 3:**
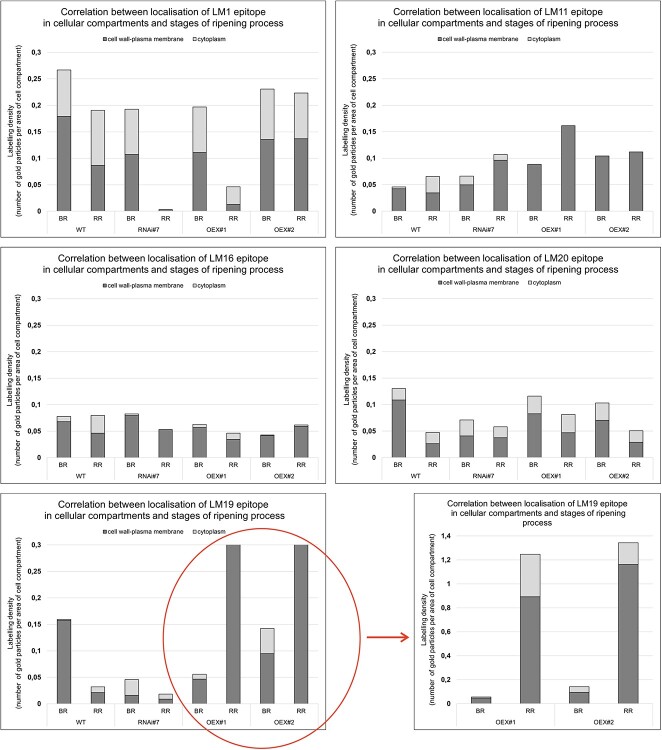
Quantitative analysis of immunogold labelling – analysis of changes in the distribution of epitopes (examined APAP1 components) in individual cellular compartments during the ripening process in the fruits of the WT plants and transgenic lines RNAi#7, OEX#1, and OEX#2.

The localization of the LM1, LM11, LM16, LM19, and LM20 epitopes analysed by immunogold labelling imaged with TEM also confirmed changes in the distribution of these epitopes during the ripening process. Detection coupled with TEM allows mapping the arrangement of particular epitopes at the subcellular level (Fig. 2). Our interpretation focused mainly on quantitative alterations in their localization in the cell wall–plasma membrane and cytoplasm compartments (Fig. 3).

Epitopes visible as red circled dots were noted in all the samples as clusters accumulated mainly in the cell wall–plasma membrane continuum. TEM imaging showed alterations in their distribution, i.e. there was an increase in the number of epitopes dispersed along the cell wall and cytoplasm in the RR stage compared to the BR stage (Fig. 2). Briefly, the comparative analyses of the examined tomato lines revealed differences in the localization of specific epitopes between the fruits of the transgenic lines and WT. In the case of extensin recognized by LM1, there are visible differences between the stage of ripening. It should be emphasized that there was a significant decrease in the LM1 epitope content in transgenic lines in the RR stage, both in the cell wall and in the cytoplasm compared to WT. In the case of xylan recognized by LM11, there is a clear accumulation of epitopes in the cell walls in OEX lines. Subcellular detection of RG-I recognized by LM16 showed no changes. However, completely different images were detected in the case of homogalacturonans recognized by LM19 and LM20. LM19 epitopes formed irregular aggregates but were mainly observed in the cell wall and middle lamella. Interestingly, in the tissue of the OEX lines, the mapping indicated a higher content of dots in comparison to WT and showed agglomerates over the entire surface of the cells. During the microscopic observations of LM20 epitopes, it was difficult to note parts of the cell without labelled HG. Also, abundant dispersion throughout the cell was correlated with the progress of the ripening process (Fig. 2).

To evaluate the mentioned changes in more detail, a quantitative analysis was performed (Fig. 3). The immunogold labelling with the LM1 antibody identified epitopes within the cell wall–plasma membrane and in the cytoplasm. In the case of WT, a 30% decrease in the number of extensin epitopes was observed during the ripening process with a simultaneous increase in their presence in the cytoplasm. Moreover, in the fruit with the silenced expression of the *SlP4H3* gene, no LM1 epitopes were observed at any cellular compartments in the RR stage. In turn, the analyses of the OEX#1 fruits showed that almost 80% of LM1 epitopes underwent degradation during the ripening process. However, in the OEX#2 line, there were no significant differences in the number of labelled epitopes in the examined compartments in both ripening stages.

The subcellular analysis performed by immunogold labelling demonstrated the presence of LM11 epitopes mainly within the cell wall. In the case of WT, an increase in the number of epitopes in the cytoplasm in the last stage of ripening associated with the cell wall degradation process was found. Interestingly, the results of the immunogold analysis of the lines with the overexpression of the *SlP4H3* gene revealed the absence of the LM11 epitope in the cytoplasm in the ripening process. Besides, compared to WT, a higher number of LM11 epitopes were identified in the case of all transgenic lines in the BR and RR stages. The results obtained in the RR stage showed an increase in the total number of labelled LM11 epitopes (40% in WT, 60% in RNAi#7, 80% in OEX#1, and 10% in OEX#2).

The immunogold labelling with the LM16 antibody confirmed that epitopes RG-I dispersed along the cell wall–plasma membrane continuum. In the WT fruits, the progress of the ripening process was accompanied by a significant increase in the number of LM16 epitopes in the cytoplasm (from 10% to 43% labelling density). The analyses of the transgenic lines did not show such a significant increase in the proportion of RG-I epitopes in the cytoplasm in the last stage of ripening. In the fruits with the silenced expression of the *SlP4H3* gene, the overall quantity of RG-I epitopes was not different from that in WT and no LM16 epitopes were observed in the cytoplasm in the RR stage. The result of the lines with the overexpression of the *SlP4H3* gene was interesting, as the total number of labelled LM16 epitopes decreased on average by 40% in comparison to WT.

The subcellular analysis of the distribution of the LM19 epitopes showed their presence in the whole area of the cell wall with a low number in the cytoplasm. In WT, a significant decrease in the number of labelled gold particles was observed with the progress of the ripening process. The results obtained in the fruit with the overexpression of the *SlP4H3* gene are interesting, as a significant increase in the number of LM19 epitopes was observed in the last stages of ripening compared to the BR stages (10-fold and 24-fold in OEX#1 and OEX#2, respectively) and the WT fruits.

The immunogold labelling with the LM20 antibody showed a similar spatial pattern of epitope distribution as in the case of LM19 mAb. However, it should be added that the number of labelled LM20 epitopes was lower compared to low methyl-esterified HG. Generally, in the case of the transgenic lines and WT, a decreased number of labelled epitopes was identified in the last stage of the ripening process. Moreover, an increase in the content of labelled HG epitopes in the cytoplasm was observed in the RR stage. In contrast, the analyses of the fruits with the silenced expression of the *SlP4H3* gene in the BR stage revealed an almost 2-fold decrease in the amount of labelled epitopes in comparison to WT. In turn, compared to WT, no significant changes in the number of LM20 epitopes were detected in the fruits with the overexpression of the *SlP4H3* gene.

### Cell wall glycome profiling using ELISA

**Figure 4:**
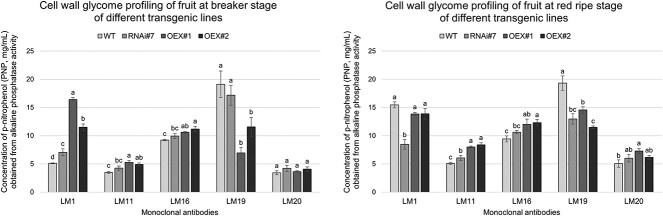
ELISA of cell wall components extracted from the fruits of the WT plants and transgenic lines RNAi#7, OEX#1, and OEX#2 in different ripening stages. ELISA with LM1, LM11, LM16, LM20, and LM19 antibodies. Different letters indicate significant differences amongst the WT and transgenic lines within a specific antibody (one-way ANOVA and Tuckey’s HSD *post hoc* test with *P* < 0.05).

The microscopic analyses showed differences between specific epitopes in samples representing the different transgenic lines in the BR and RR stages of fruit ripening, suggesting that the glycome profiling using the ELISA facilitated better quantitative visualization of changes in the cell wall components. The results shown in Fig. 4 indicate the concentration of p-nitrophenol (PNP, mg/ml) obtained through alkaline phosphatase activity linked to the secondary antibody recognizing different primary antibodies. Generally, numerous disorders in the content of all the analysed constituents were confirmed, underlining the different degrees of their presence in comparison to WT. The analysis of the results obtained with the LM1 antibody revealed statistical differences between the WT and transgenic lines in the BR stage. We observed an increase in bound antibodies based on an increase in alkaline phosphatase activity in OEX#1 and OEX#2 (15 and 10 mg/ml PNP, respectively), compared to 5 mg/ml PNP in the case of WT. No significant differences were identified between the samples analysed in the RR stage, except RNAi#7. The analysis of the samples in the BR and RR stages allowed us to identify an increase in the LM11 epitope content in the transgenic lines. The alkaline phosphatase activity increased from 3–5 to 5–8 mg/ml PNP. In the RG-I analysis, an increase in LM16 epitopes was observed in the transgenic lines compared to WT. However, no significant changes in the presence of LM16 epitopes were identified during the progress of the ripening process. The glycome profiling of low methyl-esterified HG identified lower LM19 epitope content in the lines with the overexpression of the *SlP4H3* gene compared to WT. This was confirmed by the increase in alkaline phosphatase activity bound to antibodies from 6–11 to 11–14 mg/ml PNP. Surprisingly, as in previous *in vivo* analyses of the OEX lines, an increase in the number of LM19 labelled epitopes was observed as the ripening process progressed. Interestingly, the number of LM20 epitopes was lower than that of LM19 epitopes. The statistical analysis of the presence of the LM20 epitopes at the beginning of the ripening process showed no significant differences between the transgenic lines and WT. During the ongoing ripening process, an increased number of HG epitopes was observed in the transgenic lines compared to WT.

### *In vitro* binding assay with glycome profiling

The presented assay of ‘*in vitro* reaction’ between particular components of the cell wall and isolated AGPs from tomato fruits allows analyses of the potential binding between the components in the *in vitro* environment. The immunoprinting on nitrocellulose membranes shown in Table 1 demonstrates changes in the reaction intensity representing the presence or absence of possible binding. Overall, a strong signal of the immunoblotting reaction was observed for the binding of AGPs to RG-I and cellulose. In the case of binding with RG-I, only the reaction with AGPs isolated from RNAi#7 was slightly less intensive. In the other cases, the strongest signal was recorded for AGPs isolated from the WT and transgenic lines, regardless of the stage of ripening. In the case of binding between cellulose and AGPs isolated from the WT and RNAi#7 line, the reaction indicates high signal intensity. An exception was the samples from the OEX# fruit lines where there was a low-intensity reaction.

**Table 1:** Binding assay between commercial cell wall components (arabinogalactan, rhamnogalacturonan, xylan, xyloglucan, cellulose) and AGPs isolated from the fruits of WT plants and transgenic lines RNAi#7, OEX#1, and OEX#2 in different ripening stages

		Arabinogalactan (mg/ml)			Rhamnogalacturonan (mg/ml)			Xylan (mg/ml)			Xyloglucan (mg/ml)			Cellulose (mg/ml)		
		5	10	20	5	10	20	5	10	20	5	10	20	5	10	20
		−	±	±	+	+	+	−	−	−	±	±	±	+	+	+
WT	BR	−	−	−	+	+	+	−	−	−	−	−	−	+	+	+
	RR	−	±	+	±	±	±	−	−	−	±	±	+	+	+	+
RNAi#7	BR	−	−	−	+	+	+	−	−	−	−	−	−	+	+	+
	RR	−	−	−	+	+	+	−	−	−	−	−	−	+	+	+
OEX#1	BR	−	−	−	+	+	+	−	−	−	−	−	−	+	+	+
	RR	−	−	−	+	+	+	−	−	−	−	−	−	±	±	±
OEX#2	BR	−	−	−	+	+	+	−	−	−	−	−	−	±	±	±
	RR	−	−	−	+	+	+	−	−	−	−	−	−	±	±	±

Immunoprinting on nitrocellulose membrane using the JIM13 antibody. Abbreviations: BR, Breaker stage; RR, Red Ripe stage; ‘+’, high signal intensity; ‘±’, low signal intensity; ‘–’, no signal.

The analysis of the AGP binding to commercial cell wall components showed that xyloglucan and xylan did not yield an immunoprinting reaction signal in the transgenic lines and WT. Interestingly, the use of AGPs isolated from the fruits in different stages of ripening had no significant effect on the presence of reactions in the *in vitro* binding assay. It should be added that the increased concentrations of the commercial cell wall components had no effect on the binding with AGPs either.

**Figure 5:**
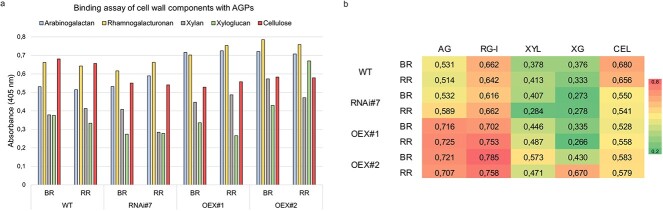
Binding assay between commercial cell wall components (arabinogalactan, rhamnogalacturonan I, xylan, xyloglucan, cellulose) and AGPs isolated from the fruits of the WT plants and transgenic lines RNAi#7, OEX#1, and OEX#2 (a) in different ripening stages. Glycome profiling with ELISA using JIM13 antibody. Heat map analysis of binding of cell wall components with AGPs (b). The numerical value in the heat map is the level of absorbance at 405 nm. Abbreviations: BR, Breaker stage; RR, Red Ripe stage; AG, arabinogalactan; RG-I, rhamnogalacturonan I; XYL, xylan; XG, xyloglucan; CEL, cellulose.

The subsequent binding assay performed using the glycome profiling approach confirmed the ability of AGPs to bind to the cell wall components (Fig. 5a). The level of absorbance demonstrates the presence/absence of binding between AGPs and other cell wall components *in vitro*. Interestingly, the analysis again showed binding between AGPs and RG-I, cellulose, and arabinogalactan. The presence of binding of AGPs with the analysed polysaccharides was represented as a heat map (Fig. 5b), which helped to find differences between particular AGPs isolated from the WT and transgenic lines and two distinct ripening stages. The analysis of the AGP binding to arabinogalactan showed an increase in absorbance in the transgenic lines with the overexpression of the *SlP4H3* gene. Similar results were obtained in the analysis of binding to RG-I. In both RG-I and AG, the level of absorbance in the line with the silenced expression of the *SlP4H3* gene was similar to that in WT. In the case of the reaction between AGPs and cellulose, a decrease in binding was found in the transgenic lines, as the highest level of absorbance was recorded in WT.

**Figure 6:**
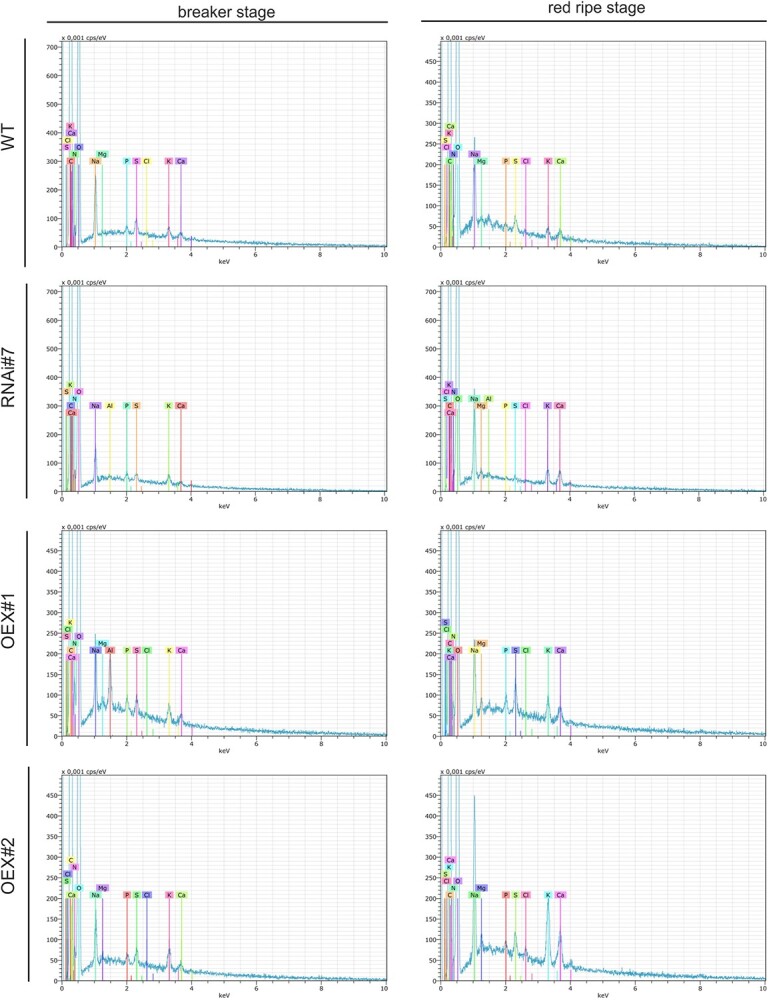
The spectral plots for the elemental composition of WT, RNAi#7, OEX#1, and OEX#2 tomato fruits.

The lowest level of absorbance was determined for AGP binding with xylan and xyloglucan. This analysis did not identify significant differences in the binding of AGPs isolated from the different ripening stages.

### Calcium amount at the tissue level

The analysis of calcium in the tomato tissue revealed a statistically significant increase in calcium ions during ripening in all the transgenic lines and WT (Fig. 6). Compared to WT, higher content of calcium-normalized mass was observed in the transgenic lines with the progress of the ripening process. The representative spectral plots helped to conduct an elemental composition analysis directed at estimation of the total amount of calcium (Fig. 6). In the case of the WT fruits, two EDS spectra at different peak levels of the voltage range of elemental calcium were observed. In the BR stage, the first one was the peak level of 400 × 0.001 cps/eV and the other one was located at 50 × 0.001 cps/eV. Also, two peaks at the levels of 300 × 0.001 cps/eV and 50 × 0.001 cps/eV were observed in the RR stage. A very similar EDS spectrum with two peaks for calcium was also observed in the tissue of the RNAi#7 line, but the peak levels were slightly lower in both BR and RR stages. However, the spectra in the OEX#1 and OEX#2 lines were the same in the BR and RR stages. Moreover, the peak levels were noted at 200 × 0.001 cps/eV and 50 × 0.001 cps/eV, significantly discriminating between the overexpressed line and the WT tissue.

**Figure 7:**
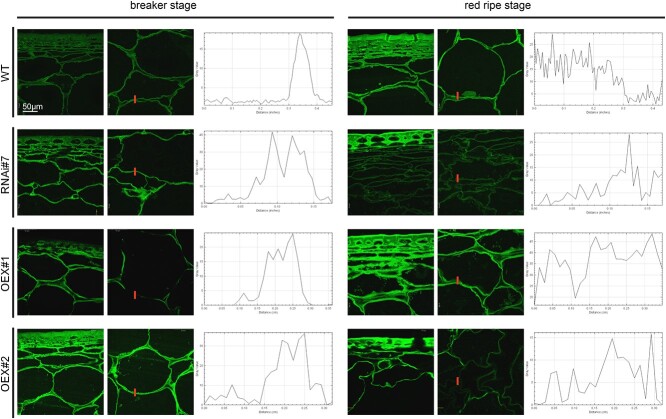
The calcium arrangement in the cell wall assembly of the fruits of the WT plants and transgenic lines RNAi#7, OEX#1, and OEX#2 in different ripening stages. Labelling with the Fluo-3 AM indicator imaged with CLSM with plots of the grey value profile (marked with a red line).

By staining with the Fluo-3 AM indicator, the arrangement of calcium ions in the fruit tissue was visualized and the cellular changes occurring during the ripening process were determined (Fig. 7). The analysis of the tissues from all the transgenic lines and WT showed an increase in fluorescence intensity with the progress of the ripening process. High amounts of calcium ions were detected in epidermal layer cells in the RR stages in all the examined lines, compared to the BR stages. It should also be added that the fluorescence intensity in the fruit tissue of the lines with the overexpression of the *SlP4H3* gene was higher than in the WT and RNAi#7 line.

The results obtained allowed us to confirm the previously presented data showing that the ripening process was accompanied by changes in the distribution of calcium ions in the cell wall. The prepared profiles are plots of the grey values of pixels along a line drawn across the cells that measure the intensity of calcium arrangement. The analyses of the profiles across the cell wall from the tomato fruits showed a significant increase in fluorescence intensity within the border of the cell wall–plasma membrane continuum. The higher fluorescence signal at the border of the cell wall was visible as one peak in the tissue of the WT and transgenic lines in the BR stage. As is well known, the ripening process disrupts the continuity of the cell wall. Analyses of calcium distribution also allow finding structural changes, which are confirmed by the lack of a single characteristic fluorescence peak. In the RR stage, the plots of the grey value profile from the labelled cell walls were featured as a disturbed pattern of numerous peaks along the whole cell walls.

## Discussion

The complex ripening process is inextricably linked with structural, morphological, and functional modifications of fruit tissue. Genetically programmed changes during ripening include hormonal and ethylene regulations, cell wall loosening, polysaccharide disruption, and pigment and aroma development [2]. Both the interactions between cell wall components and their spatio-temporal distribution have an impact on the proper functioning of the whole cell assembly [70]. The progress of ripening is linked with a lower cell-to-cell adhesion and changes in the cell wall mechanical properties [33, 71, 72]. The degradation of polysaccharides and linkages between the particular components cause softening of the cell wall [1, 49, 73]. These changes mainly result in the loss of fruit firmness [17].

Given the numerous studies on the cell wall in the successful course of the ripening process, in the current paper, we intended to determine the effect of the lack of one cell wall component on its assembly, which in turn affects the progress of the process. Thus, tomato fruits with altered *SlP4H3* expression and changed AGP content and structure were used as the research material. Detailed studies on AGP modifications in tomato fruits were reported in our previous paper [67]. In the current paper, we focused on other constituents of the APAP1.

The starting point in our analysis was the finding of clear morphological differences between the fruits of the WT and transgenic lines. Regardless of whether we analysed the silencing or overexpression of *SlP4H3*, the progress of the ripening process resulted in a different appearance of the fruit tissue. This gives grounds for concluding that the structure of the cell wall changed, and the progression of processes related to ripening deviated from the native process. The literature provides ample information about the composition of the cell wall and changes caused by fruit ripening. In the analysis of *Lycium barbarum* L. carried out by Liu and coworkers, the content of cell wall polysaccharide components decreased during the ripening process. This caused changes in the structure of the cell wall with intercellular adhesion and the resultant deformation of the parenchyma tissue [74]. Other studies carried out by Rosli and coworkers also confirmed decreased amounts of polysaccharides during strawberry ripening [73]. In summary, the reduction of cell wall components causes the disappearance of adequate support for the fruit tissues, which leads to the progression of softening [4, 74]. Other detailed analyses allowed the conclusion that changes in the amount of cellulose and hemicellulose have no significant effect on softening during the ripening process, and mainly the amount of pectins and the degree of their depolymerization and solubilization had an effect on the modification of the cell wall during the ongoing strawberry ripening [29, 73]. The data mentioned above were also confirmed in our work. The *in situ* and *ex situ* immunocytochemical approaches demonstrated quantitative and qualitative changes in the cell wall composition during the ripening process. A higher number of analysed epitopes was detected in the BR stage than in the RR stage, confirming the occurrence of degradation processes during ripening. However, the modification of P4H3 had an influence on the cell wall composition, and the changes in its content correlated with the progress of ripening. Firstly, the most pronounced disturbances were observed in the case of HG. In-depth molecular analyses showed that low methyl-esterified HG is the most distinctive component in the analysed tomato fruit lines at the beginning and end of the examined process. Low methyl-esterified HG (LM19) was present in much higher amounts than high methyl-esterified HG (LM20). Moreover, the ELISA, CLSM, and TEM analyses revealed that low methyl-esterified HG was characterized by longer persistence (RR stage) in the cell walls of the transgenic lines in comparison to WT. Especially the results of the OEX lines indicate that low methyl-esterified HG undergoes a slower degradation process with the progress of ripening. As is known, low methyl-esterified HGs are associated with the binding of calcium ions; thus, the obtained data indicated the need to perform an analysis of the content of calcium ions in the analysed tissues.

Subsequently, compared to WT, the cell walls of the transgenic lines had modified spatio-temporal distribution of particular components. *In situ* analyses were performed to verify these changes during the ripening process. An immunofluorescence analysis carried out by Ning and coworkers demonstrated changes in pectin localization during the banana fruit (*Musa* spp.) ripening process. The JIM5 (recognizes partially methyl-esterified HG (up to 40%)) and JIM7 (recognizes methyl-esterified HG (40%–80%)) antibodies used in the study increased slightly with the development of the fruits. In the case of JIM7, the signal of immunofluorescence became stronger during the development and was higher at harvest. However, after ripening, the signal significantly decreased. It was also found that the amount of LM18 antibody epitopes (which recognizes low methyl-esterified HG) was higher than that of JIM15 and JIM7 [25]. Recent evidence in strawberries and tomatoes suggests that pectin disassembly is a key factor in textural changes [22, 75]. A study on the abscission zone (AZ) during olive fruit abscission (*Olea europaea* L.) found that AZ cell separation was linked with the decreased level of JIM19 epitopes (extensin). Moreover, JIM19 labelling was present in the cell wall and the cytoplasm and absent in the cell junction regions [38]. In the present study, we found an increase in immunofluorescence intensity in tissues of the lines with the overexpression of the *SlP4H3* gene. LM19 epitopes were mainly present in the corners and cellular junctions in the BR stage, changing the location to the intercellular area in the RR stage. The location of another HG epitope, LM20, seemed to be interesting, as it was densely distributed in the cellular junctions, but the signal was significantly lower than in the case of LM19. The ripening process mainly did not affect the distribution of this epitope. Nevertheless, in the lines with the overexpression of the *SlP4H3* gene, we detected the occurrence of single conglomerates in the RR stage. In the current study, the increased *SlP4H3* expression also affected extensin distribution causing increased signal intensity in the cell wall. Extensins were visible as large conglomerates in the cytoplasm compartments, but they were absent in WT. During the progress of the ripening process, a decrease in the intensity of LM1 epitopes was determined.

Another unique disorder was noted in the microscopic observations of xylan recognized by LM11. The analyses at the subcellular level demonstrated that the LM11 epitopes in both overexpression lines were distributed mainly in the cell wall–plasma membrane. In the WT fruits in the RR stage, the examined epitopes were noted also in the cytoplasm. In the modified fruits, no such localization of xylan was observed. Furthermore, in our study, a 20%–30% increase in Xyl and a 10% increase in RG-I were determined in the transgenic lines. We may conclude that this result also proves a disturbance in the native degradation process of cell walls. An explanation can be found in available studies in which the use of mutants with the disrupted synthesis of different cell wall components made it possible to determine the role of the cell wall components in cell structure and function. Studies conducted by Biswal and coworkers showed an accumulation of HG and RG-I in the cell wall upon overexpression of the *PtGAUT12.1* gene in *Populus deltoides*. Moreover, the analyses of the *PtGAUT12.1* gene also showed a 14%–20% increase in Xyl and a 12%–17% increase in GalA. This process was accompanied by a reduction in the amount of galactose (Gal), Man, and Glc. The study also found that the tested polymers were distributed more tightly in the cell walls, which affected the plant height [76]. The study carried out by Peña and coworkers presented a modified xylan localization and distribution pattern in *Arabidopsis thaliana irregular xylem8 (irx8)* mutant, which had an impact on the morphology of plant tissue [77, 78]. In addition, the new xylan distribution pattern exerted an effect on the interactions between xylan and pectin polymers [79]. Thus, results obtained suggested that changes in expression of *SlP4H3* gene may affect other cell wall components. This is most likely due to the resulting disruption in the amount and function of AGPs. Fragkostefanakis and coworkers determined the effect of modifying P4Hs activity on the distribution of xylan and pectins in leaf epidermal cells [80].

*SlP4H1, SlP4H7,* and *SlP4H9* gene silencing was associated with a decreased amount of the JIM8 epitope (AGP) as well as the JIM11 epitope (extensin) in the case of the last two genes [80]. A study on *S. lycopersicum* presented changes in the morphology of leaf epidermal cells probably as a result of the modification of the xyloglucan and pectin epitope distribution. In addition, the results obtained are most likely related to changes in AGPs and extensins because they are covalently linked to other cell wall polymers [80]. The results obtained by Nibbering and coworkers indicate that AGPs may affect cell wall matrix properties [81], and defects in AGP synthesis may cause changes in the structure and function of the cell wall [82]. Therefore, the use of GH43 loss-of-function (the glycosyl hydrolase 43 family) mutants of *A. thaliana (gh43null)* allowed determination of the contents of cellulose, hemicellulose, and pectic sugars during AGP changes. The amount of these polymers was similar in *gh43null* and WT, but there were changes in their distribution and localization. In addition, increased signal intensity of the CoMMP microarray was observed for the pectin (LM5), xyloglucan (LM25), and extensin (JIM20) antibodies. These results indicated that the defect caused by the loss of GH43 activity had an impact on the abundance of cell wall-associated AGPs and on the content of other polymers [81]. Our previous analyses also confirmed the presence of a different amount of AGPs in fruit of lines with overexpression and silencing of the *SlP4H3* gene compared to WT [67]. The analyses of the overexpression of the *SlP4H3* gene showed that the increased P4H3 enzyme activity resulted in a higher number of AGP epitopes [67]. In the current research, we observed a disorder in the amount of all the epitopes tested in the lines with the modified *SlP4H3* gene. This correlation persisted during the ripening process. Therefore, it can be hypothesized that changes in the quantity and quality of AGPs affect other cell wall components. Mentioned disorders can disrupt fruit ripening. A great example of this might be the *Cnr* mutant, in which the aberrant localization of polysaccharide domains occurred in the cell wall, but cell wall swelling did not occur during the ripening process. In addition, in the mutant cells, there was no degradation process that takes place during proper ripening. It can be concluded that *Cnr* mutants exhibited a blocked progress of the fruit ripening process [31]. The complex structure of the cell wall with high interactions between its components is important for maintaining tissue firmness, and changing their binding ability can induce irreversible changes [29, 45, 48].

The second step of the work was to determine whether AGP from the modified fruits could be related to *ex situ* binding to other constituents. Nibbering and coworkers put forward a similar theory that APAP1 and/or cell wall-bound AGPs are targets of GH43 activity, and their modification changes the structure of the cell wall [81]. The APAP1 is a proteoglycan in which AGP binds to matrix polysaccharides [45]. Thus, the structure and function of the cell wall are affected by the present polymers and the bonds between them. However, the complete spectrum of binding is not definitively known [45, 48]. To determine the effect of the modifications of the APAP1 core protein on the whole cell wall structure, *SALK_070113c/apapap1–3* and *SALK_002144/apapap1–4* mutants were created. Using the glycome profiling method, a 5- to 83-fold increase in RG-I and HG epitopes and a 9- to 49-fold increase in xylan, compared to WT, were determined. Also, higher amounts of Rha and GalA were detected in addition to the increase in pectin and xylan in mutant extracts. Moreover, the analyses indicated less tight binding of these polymers to the cell wall due to the absence of the core protein [45]. Taking into account previous analyses and the hypothesis that AGPs have the ability to bind with other polymers, we conducted *in vitro* binding tests of extracted AGPs and commercial cell wall components. Our results indicated the ability of AGPs to bind with RG-I, AG, and cellulose. AGPs were found to have the lowest binding affinity to xylan. This was most likely related to the ability of xylan to bind to RG-I, but not AGPs, in the APAP1, as demonstrated by Tan and coworkers [45]. Whilst comparing the results obtained from the AGPs extracted from the fruits of the transgenic lines and WT, we detected stronger binding to RG-I and AG in the case of AGPs extracted from the overexpression lines. The analysis confirmed the AGP binding to AG and RG-I as showed that the intensity of the immunoreactions was higher than in the case of AGPs extracted from WT. Our data are in line with a study carried out by Hijazi and coworkers [83]. The authors also found *in vitro* interactions between AGP31 and commercial saccharides, mainly RG-I, cellulose, AG, and xylan. Hence, we can hypothesize that the APAP1 is a proteoglycan whose undisturbed structure allows proper mechanisms to take place in the cells.

The last phase of our work was to assess the content of calcium ions in the examined fruits. It is well known that pectins and AGPs have the ability to bind Ca^2+^ and form intramolecular and intermolecular calcium bridges [48, 53–56]. Research performed by Ajayi and coworkers in *glcat14* mutants showed a ~50% reduction in the amount of GlcA with a corresponding increase in galactose content and a significant reduction in calcium content [60]. Another study performed by Lopez-Hermandez and coworkers showed that glucuronidation of AG polysaccharides had an effect on the AGP-Ca^2+^ interaction [53]. The triple mutants (*glcat14a/b/d* and *glcat14a/b/e*) exhibited a significant decrease in the glucuronidation level, compared to the single and double mutants. Consequently, this had an impact on Ca^2+^ binding, as an 80% reduction in Ca^2+^ binding in triple mutants was shown, in contrast to WT. Also, in the case of the double and triple mutants, the authors observed numerous developmental and morphological defects [53]. Considering the information about the ability of pectins to bind Ca^2+^ [56] and the results of the study, the authors proposed a theory that GlcA, which bind AGPs and pectins in the APAP1, have an effect on cell wall integrity and cell-to-cell signalling [53]. In our study, we determined an increase in the calcium content during the ripening process, which was similar to the results obtained by Hyodo and coworkers. Their observations performed with the use of quantitative imaging by secondary ion-microprobe mass spectrometry (SIMS) confirmed the presence of Ca^2+^ bound by pectins in tomato fruits [29]. In all the samples of the transgenic lines and WT, the amount of calcium was higher in the RR stage compared to the beginning of the ripening process. However, the analysis of the calcium content in the cell wall of the transgenic lines confirmed the increase in the amount of this element, in comparison to WT. The SEM-EDS analysis allowed a conclusion that, in the overexpression lines, the changes in the calcium distribution as a result of ripening progress were less visible, and signals indicating calcium occurrence were observed in the whole cell surface. Given our earlier results on higher content of HG in modified lines, we may conclude that it is related to the binding affinity between calcium and pectins. An explanation of the obtained data may be provided by the information published in previous reports, in which the changes in the content of pectins and AGPs had a direct effect on the amount and distribution of Ca^2+^ ions in plant cells [57, 84].

Fruit ripening is a developmental programme strictly regulated by hormones, mainly ethylene as well as abscisic acid, jasmonic acid, and brassinosteroids [85–90]. An *Arabidopsis* P4H3 might be implicated in the oxygen-sensing pathway, and its function in plants under low oxygen has been examined [91]. In turn, oxygen sensing is coordinated by the regulation of ERF protein stability (Ethylene Response Factor) cluster of transcription factors [92, 93]. Moreover, several AGPs are upregulated during fruit ripening in tomato [7, 40, 44] suggesting putative regulation by climacteric ethylene. In this context, changes in the molecular properties of AGPs as a result of modification in P4H3 activities are associated with the assembly of the entire fruit cell wall, and we assume that the molecular changes in the cell walls may be regulated by ethylene through changes in the organization of the individual components. Such observations outline further research perspectives aimed at determining a pathway for subsequent changes in fruit metabolism and structure along with all factors that influence the fruit pericarp tissue. Correlations between oxygen concentration, ethylene level, ripening stage, P4H3 activity, AGP changes, and disruption in APAP1 organization are completely new and require extensive research.

## Conclusion

Overall, these results support the hypothesis that AGPs and pectins are responsible for the proper progression of the physiological ripening process occurring in fruits. Disorders in their quantity, structure, and temporal–spatial distribution can disrupt fruit ripening.

Here, we presumed the following order of events in response to changed *SlP4H3* expression: (1) Disruption of the AGP molecular structure causes changes in their localization in the cell wall–plasma membrane continuum [67]. Alterations in spatio-temporal AGP distribution affect the creation of connections between AGPs and other cell wall components that are included in the APAP1. (2) The disorder in the typical presence of low methyl-esterified homogalacturonan affects the presence of calcium ions in the cell wall compartments. (3) In turn, the modified structure of the cell wall matrix induces morphological variations during the progression of the ripening process.

## Material

Tomato plants (*Solanum lycopersicum* cv. ‘Ailsa Craig’) were grown in a greenhouse (Greece). The objects of the study were fruits in different stages of the ripening process. The ripening stages were classified by alternations in fruit colour [94, 95]. The first stage is Breaker (BR)—tomatoes have a pale-green colour. The next stage is Turning (TU) when tomatoes have a pale-pink colour on 10%–30% of the surface, and Pink (PINK) when 60% of the surface of tomatoes has a red colour. The last stage is Red Ripe (RR) when the entire fruits are red [95].

In the present study, we used transgenic lines with modified expression of the *SlP4H3* gene [68]. Lines with the overexpression of the *SlP4H3* gene (OEX#1 and OEX#2), which are characterized by increased activity of the P4H3 enzyme, were studied. For contrast, a line with silenced *SlP4H3* gene expression (RNAi#7), which is characterized by significantly decreased activity of the P4H3 enzyme, was analysed. A detailed description of the P4H3 effect on AGP structure was studied in our previous work [67]. Moreover, preliminary experiments suggested that there are differences in the postharvest behaviour of tomato fruits from the *SlP4H3* RNAi#7, OEX#1, and OEX#2 lines. Fruits were harvested at the Breaker+3 days and stored at 23°C for 1 month. A higher degree of fruit epidermis shrivelling was observed for both the overexpression and silencing lines whilst the softening seemed to be delayed compared to the wild type (WT). However, these experiments need to be further validated using a texture analyser.

## Methods

### Immunofluorescence labelling and CLSM imaging

The immunofluorescence method is used to evaluate changes in the cellular localization of APAP1 components in fruits in different ripening stages. Monoclonal antibodies recognizing extensin (LM1), xylan (LM11), RG-I (LM16), low methyl-esterified HG (LM19), and high methyl-esterified HG (LM20) epitopes were selected in this work. Samples were prepared according to the protocol described in our previous paper [96], i.e. fixation in paraformaldehyde (2%, Sigma, USA) and glutaraldehyde (2.5%, Sigma, USA), dehydration in a graded series of ethanol solutions, polymerization in LR White (Sigma, USA), and cutting into 1-μm-thin sections using a ultramicrotome (PowerTome XL, RMC Boeckeler, USA). The semi-thin sections were placed on poly-L-lysine–coated glass slides (Sigma Aldrich, USA) and preincubated using bovine serum albumin (2% BSA; Sigma, USA) for 30 minutes at room temperature (RT). After the washing steps, the sections were incubated with the primary antibody (diluted 1:50; Goat Anti-Rat-IgM, Kerafast, USA) overnight at 4°C. The next day, after the washing step, the sections were incubated with secondary Alexa Fluor 488 antibodies (diluted 1:200; Thermo Fisher Scientific, Denmark) overnight at 4°C. Then, the sections were counterstained with Calcofluor White (Sigma Aldrich, USA). In the control reaction, labelling with the primary antibody was omitted. An Olympus BX51 CLSM microscope with software FluoView v. 5.0. (Olympus Corporation, Tokyo, Japan) was used for imaging. Figures were prepared using the CorelDrawX6.

### Immunogold labelling and TEM imaging with quantitative analysis

Immunogold labelling of APAP1 components helps to identify changes in their localization at the subcellular level during the fruit ripening process. As in the CLSM analysis, we used LM1, LM11, LM16, LM19, and LM20 antibodies. Ultrathin sections were prepared using a diamond knife-equipped ultramicrotome (PowerTome XL, RMC Boeckeler, USA) and placed on nickel grids with formwar film. Then, the grids were preincubated using 1% BSA for 30 minutes at RT, then incubated with the primary antibody (1:10) for 3 hours at 37°C as well as with the secondary antibody diluted (1:50; Anti-Rat-IgG – Gold antibody; Sigma, USA) for 1 hour at 37°C. Before microscopic analysis, the grids were stained with a 1% uracyl acetate solution and Reynold’s reagent for 10 and 7 minutes, respectively. Control reactions were carried out by omitting the primary antibody. A TEM Zeiss EM900 transmission electron microscope operating at 80-kV acceleration voltage (Carl Zeiss AG, Oberkochen, Germany) equipped with a digital camera and software ImageSP v. 1.1.2.5 was used. The quantitative analysis revealed the correlation between the number of gold particles per 1-μm^2^ area of the cell compartment. The labelling density of the gold particles on the same size micrographs (2048 × 2048 pixels square) was counted with ImageJ v. 1.51.

### Cell wall glycome profiling – ELISA test

Enzyme-linked immunosorbent assay (ELISA) is a test for qualitative and quantitative glycan epitope profiling [96]. The preprepared samples (homogenized fruit tissue in liquid nitrogen and diluted in PBS with subsequent centrifugation at 6000 rpm for 15 minutes and 15 000 rpm for 15 minutes at RT) were added to each well on a 96-well plate (NaxiSorpTM flat-bottom, Sigma-Aldrich, USA) and incubated for immobilization for 72 hours at 37°C with shaking (350 rpm). Then, the coated plate was washed with PBS and preincubated using 0.1% BSA for 1 hour at 37°C in a plate lab shaker. After the blocking and washing steps, the plate was incubated with the primary antibody (1:20) for 1 hour at 37°C and with the secondary antibody, Anti-Rat-IgG conjugated with alkaline phosphatase (AP) (Sigma-Aldrich, USA) (1:500) for 1 hour at 37°C. The enzymatic reaction was run in the dark using a solution of p-nitrophenol phosphate (PNPP; Thermo Scientific). The absorbance at 405 nm was measured using an ELISA reader (MPP-96 Photometer, Biosan) and analysed with Statistica tools (Statistica v.13; TIBCO Software Inc. USA). Alkaline phosphatase activity was determined by the release of p-nitrophenol (PNP; Thermo Scientific) ions from PNPP. The reaction was carried out for 10 minutes at RT and then stopped by adding 2 M NaOH. In an alkaline medium, PNP takes on a yellow colour. The amount of PNP ions released was calculated from a calibration curve with the calibration coefficient y = 0.0808x, R^2^ = 0.9854. Analysis of variance (one-way ANOVA) and Tukey's Honestly Significant Difference (HSD) *post hoc* test were used to compare the mean results. In all the analyses, the significance level was set at *P* < 0.05.

### *In vitro* binding assay – immunoprinting on the nitrocellulose membrane

The *in vitro* binding assays of AGPs extracted from the fruits with Yariv Reagent (β-GlcY; Biosupplies, Australia) with commercial cell wall components on nitrocellulose membranes were carried out according to the protocol prepared by Hijazi [83] and Moller [97]. To isolate AGPs, frozen fruit tissue was homogenized, mixed with 2% CaCl_2_, and incubated at RT for 3 hours. After the incubation step, the homogenate was centrifuged (10 000 rpm 30 minutes at RT) and the supernatant was collected. β-GlcY in 2% CaCl_2_ was added to the supernatant (an equal volume to the supernatant) and incubated at RT overnight. After the incubation step, the supernatant was centrifuged (2000 rpm 15 minutes at RT) and next the precipitate was collected. The precipitate was mixed with sodium metabisulphite and heated at 50°C to reduce the diazo linkage. The solution was dialysed (dialysis tubing, 12-kDa MW cut-off, 32 mm flat width; Sigma, USA) for 48 hours. Finally, the resulting dialysate was freeze-dried. Samples of commercial cell wall components (concentration 5–20 mg/ml) were dotted onto a preprepared nitrocellulose membrane (PVDF) (Thermo Scientific, USA) and incubated for 30 minutes at RT. In the present work, the following elements of the APAP1 were selected: arabinogalactan (Megazyme, USA), rhamnogalacturonan (Megazyme, USA), partially acetylated xylan (Megazyme, USA), xyloglucan (Megazyme, USA), and cellulose (Sigma, USA) to check the ability to bind with the isolated AGPs. After washing with Tris-buffered saline (TBST, 7.6 pH), the membrane was incubated with the solution of isolated AGPs (a concentration of 20 μg/ml was selected experimentally) overnight at 4°C. After the washing steps, the membrane was blocked using 3% BSA in TBST for 1 hour at RT. After the blocking and washing steps, the membrane was incubated with the primary antibody diluted in 1.5% BSA (1:200) for 2 hours at RT and with the secondary antibody diluted in 1.5% BSA (1:1000) for 1 hour at RT. The membrane was treated with 20 ml of AP buffer with substrates: 9 mg of nitro-blue tetrazolium (NBT; Sigma, USA) in 0.3 ml of water and 0.7 ml of N, N-dimethylformamide (DMF; Thermo Scientific, USA) and 4 mg of 5-bromo-4-chloro-3-indolylphosphate (BCiP; Sigma, USA) in 1 ml of water. Measurements and analysis of colour intensity were carried out using GelDoc Go Imaging System (Bio-Rad, USA) and Image Lab Software v. 6.1 (Bio-Rad, USA). Data for each sample were obtained from the results of three independent experiments and represented in the table as the presence and absence of binding. The measurements were classified into 3 categories: ‘+’ – high signal intensity, ‘±’ – low signal intensity, and ‘–’ – no signal.

### Binding assay and screening by ELISA

The results of the binding assay described by Biswal and coworkers [76] based on the use of glycome profiling with screening with ELISA-based monoclonal antibodies were shown in histograms and heat maps. Samples of commercial cell wall components at a concentration of 20 mg/ml were added to each well on a 96-well plate and incubated for immobilization for 72 hours at 37°C with shaking (350 rpm). Then, the coated plate was washed with PBS, and AGP solutions (concentration 20 μg/ml) were added to each well overnight at RT. After blocking with 0.1% BSA for 1 hour and washing steps, the plate was incubated with the primary antibody diluted in PBS (1:20) for 1 hour at 37°C and with the secondary antibody Anti-Rat-IgG conjugated with AP diluted in PBS (1:500) for 1 hour at 37°C. After the washing step, the enzymatic reaction was run in the dark using PNPP; the reaction was then stopped with 2 M NaOH. The absorbance at 405 nm was measured using an ELISA reader (MPP-96 Photometer, Biosan) and analysed with Microsoft tools. The heat map presenting the colour intensity is proportional to the numerical value of absorbance.

### Estimation of the calcium amount at the tissue level – SEM-EDS

The elemental composition analysis was performed with the use of the energy-dispersive X-ray microanalysis method and scanning electron microscope (SEM). The surface of the fruit sample was examined using energy-dispersive X-ray spectroscopy (EDS) with a Bruker X-ray detector equipped with an SEM with operating conditions of the electron microprobe of 20 kV (SEM, Zeiss Ultra Plus, Oberkochen, Germany). After the fixation step (2% paraformaldehyde and 2.5% glutaraldehyde), the samples were washed 2 times in PBS and dehydrated in a graded ethanol series. Next, the samples were dried in a critical point dryer using liquid CO_2_ to redraw water (CPD7501, Polaron Range, UK) and coated with gold using a sputter coater. SEM-EDS imaging allows semiquantitative measurements of the amount of calcium. Each sample had at least five replicates of X-ray line scans, and then data analysis was done as in Li and coworkers [98].

### Determination of the intracellular calcium level – Imaging with the Fluo-3 AM indicator

As in the studies performed by Li [98] and Qiu [99], the Fluo 3-AM (Sigma Aldrich, USA) indicator was used as a reagent to detect the localization of calcium at the cellular level and to estimate the different calcium content by measurement of the fluorescence intensity. Briefly, slices of the fruits were rinsed twice with dimethyl sulfoxide (DMSO; Sigma Aldrich, USA) and incubated in a working solution of Fluo 3-AM for 2 hours at 4°C in darkness. The Fluo 3-AM (1 mM in DMSO) was mixed with Pluronic F-127 (10% solution in DMSO; Thermo Fisher Scientific). Calcium detection was performed using an Olympus BX51 Confocal Laser Scanning Microscope equipped with software Fluo-View v. 5.0. (Olympus Corporation, Tokyo, Japan). The plots of the grey value profile were counted with ImageJ 1.51 software.

## Acknowledgements

This research was funded by the National Science Center, Poland (SONATA 16, grant number 2020/39/D/NZ9/00232). Also, this work has been supported by the COST Action ‘Roxy-COST’ (CA:18210) which is funded by the European Cooperation in Science & Technology. Also, work has been financed by the European Regional Development Fund of the European Union and Greek national funds through the Operational Competitiveness, Entrepreneurship and Innovation, under the call RESEARCH-CREATE-INNOVATE (project code: T2EDK-01332: n-Tomatomics - Development of new tomato cultivars by using -omics technologies).

## Author contributions

N.K. Investigation, Methodology, Data curation, Visualization, Writing—original draft; A.L. Supervision, Project administration, Funding acquisition, Formal analysis, Writing—review & editing; M.L. Investigation (TEM imaging); D.D., S.B., K.B., P.G. Investigation (created the transgenic lines); P.K. Investigation (created the transgenic lines), Writing—review & editing; and A.Z. Writing—review & editing.

## Conflict of interest statement

The authors declare that they have no conflict of interest.

## Data availability statement

The data underlying this article are available in the RepOD database [Leszczuk Agata, 2023, Studies on arabinogalactan proteins (AGPs) in fruits, file folder no A_6] at https://doi.org/10.18150/KMG7WI.
